# Construction of MicroRNA-mRNA Regulatory Network in the Molecular Mechanisms of Bleomycin-Induced Pulmonary Fibrosis

**DOI:** 10.1155/2022/7367328

**Published:** 2022-03-30

**Authors:** Liuyin Chen, Zhiling Shi, Lin Deng, Jiangtao Wu, Housheng Wang, Yushuang Lu, Ting Fan, Jiamei Lu, Weimei Huang, Kai Hu

**Affiliations:** ^1^Department of Radiation Oncology, The First Affiliated Hospital of Guangxi Medical University, Nanning, 530021 Guangxi Province, China; ^2^Department of Radiation Oncology, Nanxishan Hospital of Guangxi Zhuang Autonomous Region, Guilin, 541004 Guangxi Province, China

## Abstract

Bleomycin is a common antitumor agent used to treat many different types of malignancies; however, its main side effect is pulmonary fibrosis. The mechanism of bleomycin-induced pulmonary fibrosis (BIPF) has not been fully elucidated. Therefore, to further explore the molecular mechanisms of BIPF, we screened for microRNA (miRNA) and mRNA expression obtained from BIPF samples from the Gene Expression Omnibus database. Subsequently, we identified the differentially expressed miRNAs and genes that overlapped with the differentially expressed miRNAs target genes, predicted by using the miRWalk database selected as a candidate. The candidate genes were visualized based on Gene Ontology and the Kyoto Encyclopedia of Genes and Genomes analyses. A protein-protein interaction network was constructed. Hub differentially expressed genes were selected and corresponding miRNAs to construct a miRNA-mRNA regulation network. Then, we chose three key miRNAs to study their regulatory relationship in bleomycin-induced pulmonary fibrosis. Finally, mouse lung epithelial cells TC-1 and MLE-12 were treated with bleomycin with qPCR to validate the results of three important hub genes and all key miRNAs. And dual-luciferase report experiment was carried out to verify the interaction of mmu-miR-1946a and serpina3n. The results revealed that the imbalance of matrix metalloproteinase-9 (MMP-9) and tissue inhibitor of metalloproteinase-1 (TIMP-1) plays a pivotal role in the occurrence and development of BIPF. In addition, Serpina3n and mmu-miR-1946a were proved interaction and may be involved in the regulation of the balance between MMP-9 and TIMP-1. The experimental results also verify the analysis. Our findings provide new insights into the key mediators and pathways related to the molecular mechanisms of BIPF.

## 1. Introduction

Bleomycin (BLM), a glycopeptide antibiotic isolated from Streptomyces, acts as an antitumor agent for many different types of malignancies, including lung cancers [1]. The cure rate of metastatic testicular cancer can be as high as 90% when chemotherapy regimens contain BLM [2]. However, the main side effect of BLM is pulmonary toxicity that can cause pulmonary fibrosis in approximately 10-23% of patients [3], which greatly limits the application of this drug. Some studies have explored the mechanism of BLM-induced pulmonary fibrosis (BIPF), and the experiments of animal pulmonary fibrosis with BLM have also been applicated [4, 5]. Nevertheless, the specific mechanism of BIPF has not been clarified. The research on BIPF by bioinformatics method is rare despite the exploration of related genes is helpful to the individualized treatment of patients; the research on BIPF by bioinformatics method is still rare. Consequently, further studies are needed to find the potential approach to overcome this problem.

The interactions between mRNAs and microRNAs (miRNAs) are major regulators of pulmonary fibrosis. Xu et al. [6] demonstrated that miR-326 attenuates silica-induced pulmonary fibrosis by inhibiting inflammation and promoting autophagy by targeting TNFSF14 and PTBP1, whereas Tang et al. [7] found that SHIP-1 can act as a target for miR-155 to regulate the responses of endothelial cells in pulmonary fibrosis. Moreover, Wei et al. [8] found that miR-133a mediates transforming growth factor-*β*1, which blocks myofibroblast differentiation and pulmonary fibrosis. Although numerous miRNAs and mRNAs have therefore been found to participate in pulmonary fibrosis, the vital roles of the miRNA-mRNA regulatory network in BIPF remain unclear, which may be addressed through sequencing, bioinformatics analysis, and functional verification.

In the current study, we aimed to elucidate the interactions between miRNAs and mRNAs in a mouse model of BIPF. Our ultimate objective was to provide a theoretical basis for the prevention and alleviation of BIPF.

## 2. Materials and Methods

### 2.1. Microarray Data

**Figure 1:**
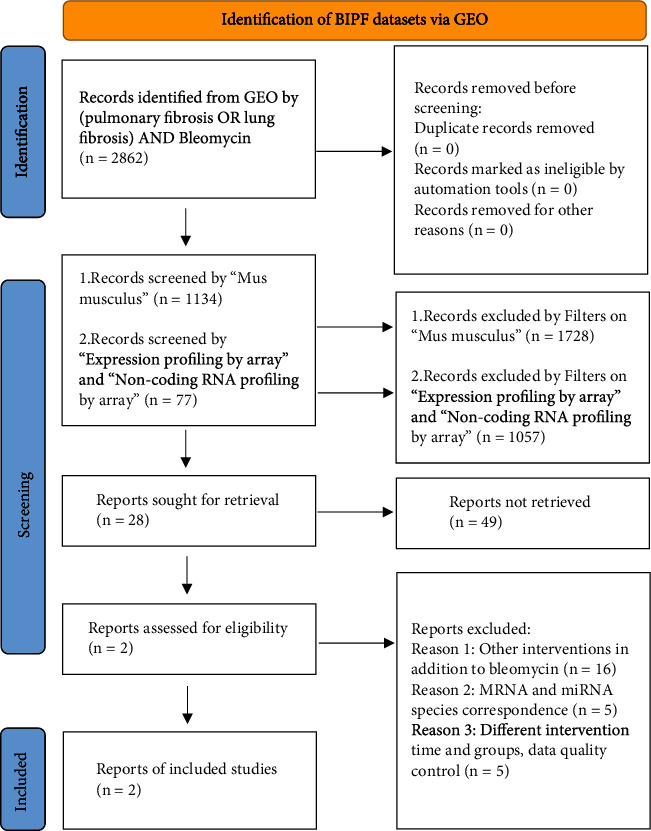
The PRISMA flow diagram of BIPF datasets in GEO.

We retrieved the database from the Gene Expression Omnibus (GEO; https://www.ncbi.nlm.nih.gov/gds), an open source for sequencing data [9, 10]. The PRISMA flow diagram about BIPF data screening was shown in Figure 1. We then focused on the target miRNA expression profile GSE155823 ([GSE155823], Sudhiranjan Gupta, 2020, Studies on bleomycin-induced lung fibrosis in mice, GEO, GSE155823) based on the GPL28959 platform (LC Sciences Mouse miRNA Array [MRA-1002_MIRMOUSE_22]) and mRNA expression profile GSE77326 ([GSE77326], Lv X and Liu S, 2017, Expression analysis of bleomycin-induced pulmonary fibrosis mouse model lung tissues, GEO, GSE77326) and matching the GPL15887 platform (Nimblegen Mouse Gene Expression Array [100718_MM9_EXP]) in GEO [11]. Both datasets were sequenced from BIPF in mice or mice treated with saline solution (negative control group). In this study, the data were downloaded from the public database for analysis, and ethical approval was not required.

### 2.2. Identification of the Differentially Expressed miRNAs (DEMs) and Genes (DEGs)

We used the RAffy package (https://bioconductor.org/packages/release/bioc/html/affy.html) to set the original chip and correct the background data by log2 for GSE155823 and GSE77326 [12]. In addition, we used the LIMMA package (https://bioconductor.org/packages/3.10/bioc/html/limma.html) in R [13]. Thresholds for analysis were adjusted *P* < 0.05, ∣log_2_ fold change (FC) | >0.585 for miRNAs and *P* < 0.01, ∣log_2_FC | >0.585 for mRNAs.

### 2.3. Predicted Target Genes of DEMs and Identified Candidate Genes

miRWalk (http://mirwalk.umm.uni-heidelberg.de) is a comprehensive miRNA target gene database that includes information on miRNA target genes in humans, mice, and other species [14] and integrates information from databases. We then entered the miRNA target genes and DEGs using Venn figure online tools (http://bioinformatics.psb.ugent.be/webtools/Venn/) to search for overlaps between the groups.

### 2.4. Function and Pathways Enrichment Analyses

After identifying the candidate genes, we analysed the function and enriched biological pathway by Gene Ontology (GO) and Kyoto Encyclopedia of Genes and Genomes (KEGG). We then evaluated GO terms of candidate genes in the Database for Annotation, Visualization, and Integrated Discovery (DAVID) (v.6.8, https://davi.ncifcrf.gov) online database [15]; statistical significance was set at *P* < 0.05. We used the R ggplot2 package (https://mirror.lzu.edu.cn/CRAN/) for the visualization of GO annotations for biological processes (BPs), cell components (CCs), and molecular functions (MFs) and the online mapping software hiplot (https://hiplot.com.cn/basic) to visualise the KEGG enrichment results.

### 2.5. Protein-Protein Interaction (PPI) Networks and Screening of Hub Genes

For the genes and proteins relationships, the STRING database (version: 11.0, https://string-db.org) was used [16] to obtain the PPI network, which calculated node degrees and provided different colours using the Cytoscape Network Analyzer (version 3.8.2). The MCODE and CytoHubba plug-ins were used to extract the top 10 hub genes to generate a subnetwork [17]. Then, DAVID and GO enrichment were used to evaluate the signalling pathways of the top 10 hub genes. Statistical significance was set at *P* < 0.05.

### 2.6. Construction of miRNA-mRNA Regulatory Network and Identification of Key miRNAs

Hub genes and corresponding miRNAs constructed the miRNA-mRNA interaction network, and the CytoHubba plug-in was then used to identify the top three key miRNAs. After which, the miRNAs were matched to the predicted target genes in miRWalk.

### 2.7. Validation of the Results by qPCR and Elisa Experiments

To verify the results of three important hub genes and the key miRNAs, we used the following cell lines: RRID: TC-1 (ATCC Cat# CRL-2785, RRID: CVCL_4699) and MLE-12 (ATCC Cat# CRL-2110, RRID: CVCL_3751), treated with BLM (1 *μ*g/ml) by conventional culture for 48 h to extract RNA and perform qPCR experiments (see Table 1).

### 2.8. Dual-Luciferase Reporter Assays

Constructed dual-luciferase reporter vectors and 293T cells (ATCC Cat# CRL-3216, RRID: CVCL_UE53) were cultured according to the above conditions. According to the instructions, the prepared DNA lip2000 complex and RNA lip2000 complex were added to the inoculated cells for cell transfection. Lysing cells for fluorescence detection. LARII (Firefly luciferase reaction working solution) and Stop & Glo® (Renilla luciferase reaction working solution) were added to detect the luciferase activity. The detection should be completed within 30 min as far as possible.

### 2.9. Statistical Analysis

The Statistical Package for Social Sciences, version 23.0 (SPSS, Inc., Chicago, USA), was used for the statistical analysis. qPCR data analysis was conducted using the *t* test (unpaired *t* test, two-tailed). Dual-luciferase reporter assays were analyzed by a one-way ANOVA (homogeneity of variance test: *P* > 0.05). Meanwhile, the bioinformatics analysis was related to computer algorithm, which did not involve the author's statistical analysis.

## 3. Results

### 3.1. Identification of DEMs and DEGs

**Figure 2:**
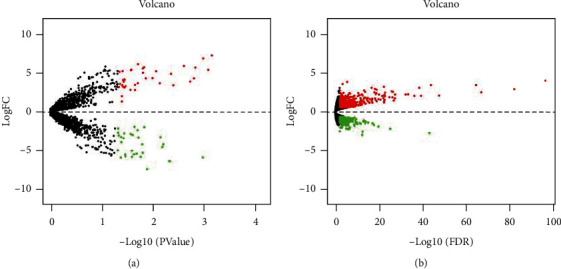
Analysis of Gene Expression Omnibus (GEO) data and cluster differentially expressed miRNAs (DEMs) and genes (DEGs). (a) Volcano plot (down- and upregulated indicated by green and red, respectively; black means nonsignificant). Adjusted *P* < 0.01, ∣log2 fold change | >0.585. DEMs: differentially expressed miRNAs. (b) Volcano plot (down- and upregulated indicated by green and red, respectively; black means nonsignificant). Adjusted *P* < 0.05, ∣log2 fold change | >0.585. DEGs: differentially expressed genes. (R LIMMA package, 3.10).

To identify DEMs and DEGs, we carried out miRNA (GSE155823) and mRNA (GSE77326) microarray analyses. In the miRNA microarray analysis, 37 miRNAs (i.e., miR-1199-3p, miR-6953-5p, and miR-6953-3p) were upregulated, and 30 miRNAs, including miR-206-3p, miR-6353, and miR-3106-3p, were downregulated (see Table 2). We then created a volcano plot based on DEMs (see Figure 2(a)). In addition, mRNA expression profiles (960 DEGs) were identified, of which 550 were upregulated and 410 downregulated (see Figure 2(b)).

### 3.2. Prediction of the Target Genes of DEMs

**Figure 3:**
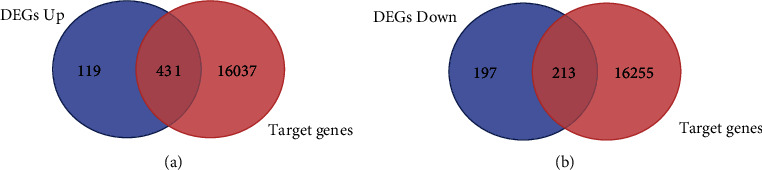
Candidate genes overlapped by target genes and DEGs with Venn diagram. (a) 431 overlapped candidate genes upregulated. (b) 213 overlapped candidate genes upregulated (miRWalk and Venn figure online tools).

We used the miRWalk database and predicted target genes of DEMs. The database identified 66 common DEMs (excluding PUC2MM in Table 2) and predicted 16,468 target genes. These target genes were then compared with DEGs, and common genes in the two groups were identified. In total, 644 overlapping genes are identified as candidate genes, of which 431 genes are upregulated and 213 downregulated (see Figure 3).

### 3.3. Function and Pathway Enrichment Analyses of Candidate Genes

**Figure 4:**
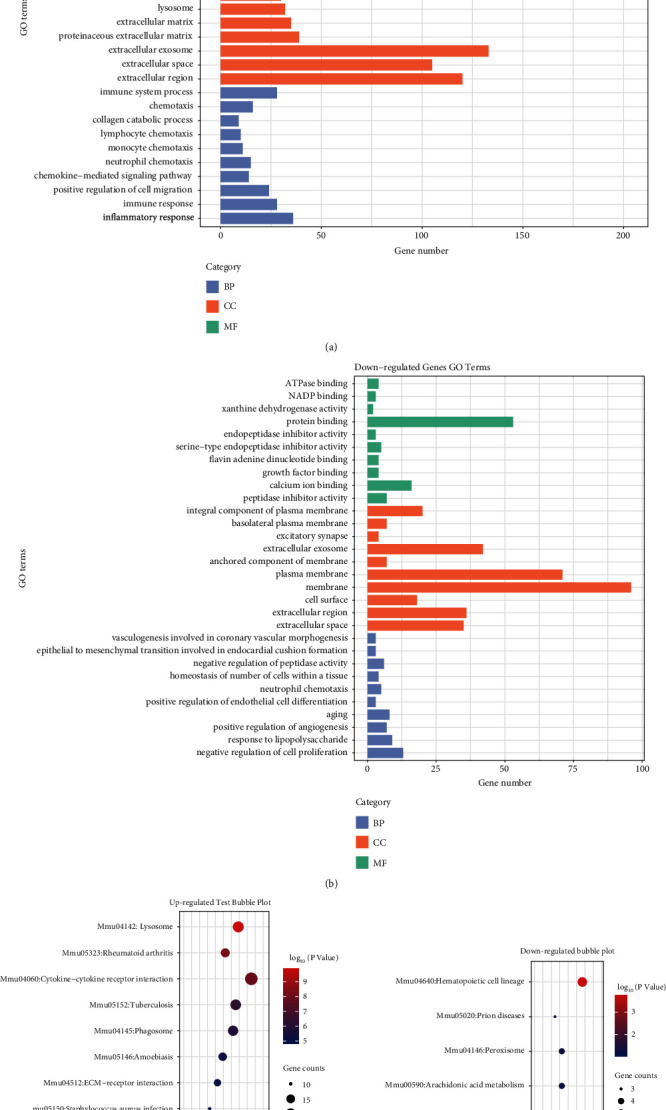
GO and KEGG enrichment analyses of candidate genes. (a) Top 10 GO terms for upregulated candidate genes. (b) All GO terms for downregulated candidate genes. (c) KEGG enrichment analysis of top 10 upregulated candidate genes. (d) KEGG enrichment analysis of all downregulated candidate genes. GO: Gene Ontology; KEGG: Kyoto Encyclopedia of Genes and Genomes (DAVID v.6.8 and hiplot).

The candidate genes, which overlapped by miRNA target genes and DEGs, were uploaded to the DAVID database. We identified a total of 44 KEGG pathways, of which 38 were upregulated (only the first 10 were selected for visualisation) and 6 were downregulated. According to the *P* values, we created a plot to visualise the GO enrichment results for the top 10 upregulated and downregulated pathways (see Figures 4(a)–4(b)). The upregulated genes in the BP category are mainly concentrated during inflammation, immune response, cell migration, and chemotaxis, whereas the downregulated genes in the BP category are concentrated in negative cell proliferation regulation, positive regulation of angiogenesis, negative regulation of peptidase activity, response to lipopolysaccharides, and aging. In the CC group, the upregulated genes were mainly concentrated in the cell membrane, extracellular region and space, exocrine body, and cell surface, whereas the downregulated genes were mainly distributed in the cell and plasma membranes, exosome, extracellular region, and extracellular space. In the MF category, the upregulated genes were enriched in calcium binding, cytokine, chemokine, metallopeptidase activities, and heparin binding, while the downregulated genes were concentrated in protein and calcium binding and peptidase inhibitor activity. In addition, KEGG analysis show that the upregulated genes are significantly enriched in the lysosome and cytokine receptor interaction pathways, whereas the downregulated genes are mainly involved in the hematopoietic pathway (see Figures 4(c)–4(d)).

### 3.4. Construction of the PPI Network and Modular Analysis Screening of Hub Genes

**Figure 5:**
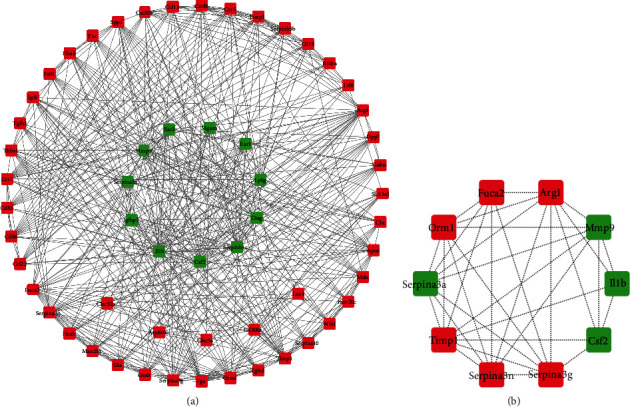
Construct of PPI networks. (a) Hub candidate genes PPI subnetwork. (b) Top 10 hub candidate genes in PPI subnetworks. Red indicates upregulated genes, and green indicates downregulated genes. PPI: protein-protein interaction (Cytoscape v3.8.2).

We constructed PPI network which included 314 upregulated and 90 downregulated genes. Subsequently, the MCODE plug-in Cytoscape (v3.8.2) was used to identify and extract hub genes, among which 46 upregulated and 11 downregulated hub genes are identified (see Figure 5(a)). The CytoHubba plug-in was then used to filter the top 10 nodes of the hub genes such as MMP-9 and TIMP-1, which are the common genes involved in pulmonary fibrosis (see Figure 5(b)). DAVID online database analysis and GO evaluation of the top 10 hub genes were carried out, and we found that the top 10 hub genes are mainly concentrated on the tumor necrosis factor (TNF) signalling pathway (see Table 3).

### 3.5. Construction of miRNA-mRNA Regulatory Network and Identification of Key miRNAs

**Figure 6:**
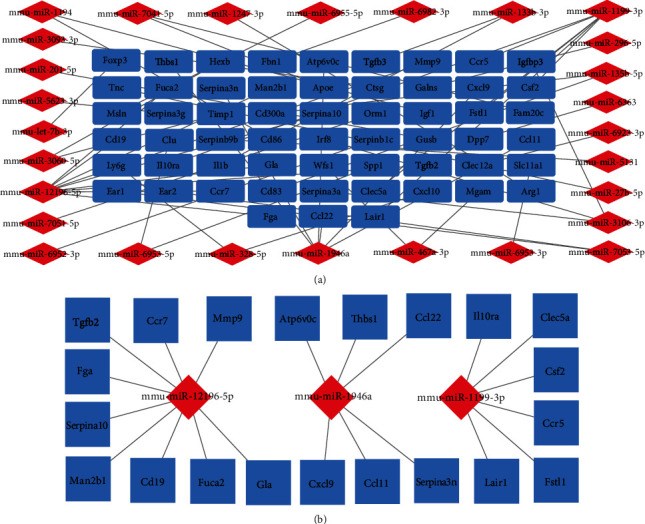
miRNA-mRNA regulatory network. (a) Red indicates miRNAs, while blue indicates mRNAs. (b) Key miRNAs network. The top three key miRNA networks are shown. Blue indicates mRNAs, and red indicates miRNAs (Cytoscape v3.8.2).

To study the regulatory relationships, we constructed a miRNA-mRNA network using 57 hub genes (46 upregulated and 11 downregulated) and 28 DEMs (16 upregulated and 12 downregulated). The network consisted of 85 nodes and 56 edges (see Figure 6(a)). The first three DEMs (mmu-miR-12196-5p, mmu-miR-1946a, and mmu-miR-1199-3p) were selected as the central miRNAs and drawn as subnets by calculating the value (see Figure 6(b)). Among them, mmu-miR-12196-5p corresponded to nine target genes (Fga, Cd19, Ccr7, Mmp9, Tgfb2, Man2b1, Gla, Fuca2, and Serpina10), mmu-miR-1946a to six (Cxcl9, Ccl22, Serpina3n, Ccl11, Atp6v0c, and Thbs1), and mmu-miR-1199-3p also to six (Lair1, Ccr5, Fstl1, Clec5a, Csf2, and Il10ra).

### 3.6. Validation of the Results by qPCR Experiments

**Figure 7:**
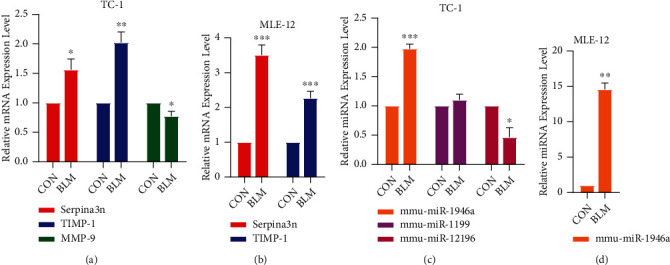
The qPCR results of three hub genes and key miRNAs in TC-1 and MLE-12. (∗ represents *P* < 0.05, ∗∗ represents *P* < 0.01, and ∗∗∗ represents *P* < 0.001; SPSS Statistics 23 and GraphPad Prism 8.0.2.263).

After treating with BLM, the qPCR results showed that the hub genes of serpina3n and TIMP-1 were upregulated, and MMP-9 was upregulated in TC-1 (see Figure 7(a)). serpina3n and TIMP-1 were upregulated in MLE-12 (see Figure 7(b), MMP-9 was not detected). It was mutual confirmation with our analysis. In the key miRNAs, mmu-miR-1946a was upregulated, and mmu-miR-12196 was down regulated in TC-1 (see Figure 7(c)). Mmu-miR-1946a was upregulated, and mmu-miR-1199 and mmu-miR-12196 were not detected in MLE-12 (see Figure 7(d)). Among them, mmu-miR-1946a was mutual confirmation with our analysis.

### 3.7. Verification of the Binding of serpina3n and Mmu-miR-1946a

**Figure 8:**
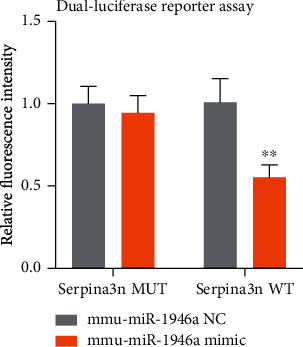
The dual-luciferase reporter assay of seprina3n and mmu-miR-1946a in 293T. (∗ represents *P* < 0.05, ∗∗ represents *P* < 0.01, and ∗∗∗ represents *P* < 0.001; SPSS Statistics 23 and GraphPad Prism 8.0.2.263).

The expression of Serpina3n and mmu-miR-1946a in MLE-12 and TC-1 was confirmed to be consistent with the bioinformatics analysis results. After that, we further discussed the binding of serpina3n and mmu-miR-1946a in tool cell 293. The dual-luciferase reporter assay results showed that both mmu-miR-1946a NC group and mimic had similar fluorescence expression of intensity in the mutant type serpina3n. On the contrary, the fluorescence expression of the mmu-miR-1946a mimic group was lower than that of the NC group in the wild-type serpina3n (see Figure 8). This result demonstrated that the wild-type serpina3n was bound with mmu-miR-1946a to degrade the fluorescein, which was confirmed the interaction between serpina3n and mmu-miR-1946a.

## 4. Discussion

In patients with malignancies treated with BLM, pulmonary fibrosis remains a major problem in clinical practice. Therefore, further work is needed to explore the molecular mechanisms and identify potential therapeutic targets of BIPF. Accordingly, in this study, we used bioinformatics analysis to provide new insights into key mediators and pathways related to the molecular mechanisms of BIPF. KEGG analyses showed enrichment pathways involving the top 10 hub genes and the top three key miRNAs were consistent with the pathophysiological processes of pulmonary fibrosis, including inflammatory and immune responses [18]. These findings suggested that the top 10 hub genes, top three key miRNAs, and their target genes obtained in this study may be involved in BIPF.

miRNAs are endogenous small RNAs that regulate gene expression after transcription [19]; these molecules mainly act on the base pairing of the 3′-untranslated region (UTR) of mRNA to posttranscriptionally regulate the mRNA [20]. Specific miRNAs have been reported to be closely associated with pulmonary fibrosis [8]. We found that mmu-miR-1946a is one of the top three key miRNAs after analysing DEMs in BIPF and normal lung tissues. In a study by Fan et al. [21], mmu-miR-1946a is predicted to bind to the transforming growth factor (TGF-*β*1) 3′ UTR in mouse lung fibroblasts, thereby inhibiting its transcription. And they also found that alcohol can suppress mmu-miR-1946a and induce TGF-*β*1, which was reported to worsen fibroproliferation following BIPF in alcohol-fed mice. These findings are consistent with the results of our study, which demonstrated that mmu-miR-1946a may play vital regulatory roles in BIPF.

In this study, among the DEGs identified between the pulmonary fibrosis and control groups, three of the top 10 hub genes (serpina3a, serpina3n, and serpina10) and two target genes of the three key miRNAs genes (serpina3n and serpina10) were serine protease inhibitor (serpina) family genes. Serpina belongs to the serpin family, which is a widely distributed protease inhibitor. Among the serpina family, the serpina3 (alias: alpha-1-antichymotrypsin, *α*-ACT) subfamily is a typical acute phase protein that is significantly upregulated in response to inflammation in the circulatory system. Moreover, serpina3 participates in inflammatory responses and connective tissue renewal [22]. However, overexpression of serpina3 affects inflammation [23]. Previous studies have shown that serpina3 is associated with malignant melanoma and other diseases [24]. Our results show that serpina3n is a potential target gene of mmu-miR-1946a, and the expression of serpina3n is upregulated as mentioned above. Although regulation of mRNA translation and stability by miRNAs is achieved by degrading mRNA in most studies, various studies found that miRNAs can activate gene transcription abnormally as an enhancer trigger [25], suggesting that miRNAs have a dual regulatory mechanism on mRNA. In this study, upregulated serpina3n is the target gene of upregulated mmu-miR-1946a, which may be a positive regulatory mechanism. The upregulated expression of mmu-miR-1946a may be achieved through the positive regulation of miRNA, but the specific regulatory mechanism remains to be confirmed. To the best of our knowledge, no reports have described to date the relationships between serpina3 and pulmonary fibrosis. The above results suggest that the serpina3 subfamily may play important roles in BIPF in mice.

**Figure 9:**
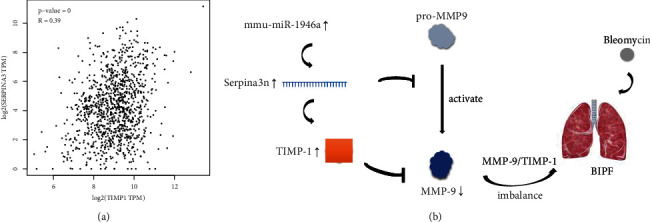
Exploration of possible mechanisms in BIPF. (a) The relationship between serpina3 and TIMP-1 was analysed by GEPIA (*P* < 0.05, *R* = 0.39). (b) Schematic diagram of possible mechanism in BIPF (Adobe Photoshop CC 2018).

In the top 10 hub genes, we found that the tissue inhibitor of metalloproteinase-1 (TIMP-1) expression was increased. TIMP-1 is a member of the metalloproteinases (TIMPs) family, with the main function of regulating the hydrolysis of cell matrix and surface proteins. TIMP-1 expression is also used as a biomarker to evaluate the prognosis of cancer patients [26] and specifically inhibit the activity of matrix metalloproteinases (MMPs). The expression of serpina3 and TIMP-1 is increased in the development of chronic obstructive pulmonary disease, which indicates that serpina3 and TIMP-1 may show the same expression trend in the disease [27], which is consistent with the relationships observed in our study. In addition, we used the gene expression profile interaction Atlas (GEPIA) database [28] for gene correlation analysis and found that the expression of serpina3 was correlated with the gene expression of TIMP-1 (see Figure 9(a)), which supported our results.

MMPs are an important family of proteases that degrade the extracellular matrix and its components. Among them, MMP-9, which exists in our top 10 hub genes, is the largest enzyme in the family [29]. MMP-9 has been found to be involved in the process of pulmonary fibrosis [30], and TIMP-1 is a specific inhibitor of MMP-9 [31]. Under normal physiological conditions, MMP-9 and TIMP-1 maintain a delicate balance in vivo, but the imbalance of the MMP-9/TIMP-1 ratio is related to the progression of many diseases. Liu et al. [32] found that Icariside II attenuates cerebral ischemia/reperfusion-induced blood-brain barrier dysfunction in rats via regulating the balance of MMP-9/TIMP-1. In the clinical study of pulmonary fibrosis, an abnormal MMP-9/TIMP-1 ratio indicates a dysfunctional matrix degradation system, which leads to the progress of pulmonary fibrosis [33]. Wu et al. [34] found that IL-33 promoted the process of pulmonary fibrosis by inducing the imbalance between MMP-9 and TIMP-1. All the above studies showed that the imbalance of the MMP-9/TIMP-1 ratio can lead to the occurrence of many diseases, especially pulmonary fibrosis. Our study further demonstrates that the expression of MMP-9 decreases and the expression of TIMP-1 increases in mice three weeks after BIPF (see Figure 9(b)). Additionally, serpina3 is a specific activator of MMP-9 [35]. a3 expression increased, while MMP-9 expression decreased. In this study, the expression of serpina3 was increased, and then, the expression of MMP-9 decreased. The expression trends of these two genes are consistent with the biological effect of serpina3, which in turn supports the findings of this study. We also verified it through experiments in vitro. Therefore, the imbalance of the MMP-9/TIMP-1 is involved in the disease progression of BIPF.

From the internal threat to the validity of this study, we draw a conclusion through a large number of scientific and recognized approaches to screened and analyzed data. And then, we verified the results through in vitro experiments, so it contains a fairly high availability. However, the threat of our research is that we did not verify the results using functional experiments in vivo, and the effectiveness needs to be further performed in animal models. From the external threat to the validity, our validation experiment in vitro had been repeatedly verified, and the same trend and results had been obtained three times. So, it is also scalable for other similar or identical studies. Despite its limitation, this study is the first to use bioinformatics analysis to explore the hub genes and key miRNAs related to BIPF from the perspective of miRNA-mRNA regulatory network and the possible molecular mechanism to the best of our knowledge. Therefore, in consideration of our research findings being preliminary, we will continue to explore further mechanisms in the future.

## 5. Conclusions

In conclusion, our results indicate that the imbalance of MMP-9/TIMP-1 plays a critical role in BIPF in mice. Serpina3 and mmu-miR-1946a may be involved in the regulation of MMP-9 and TIMP-1. Our findings provide new insights into key mediators and pathways related to the molecular mechanisms in BIPF.

## Figures and Tables

**Table 1:** Primer sequences of three important hub genes and the key miRNAs.

Gene	Primer sequence (5′ → 3′)
serpina3nF	GCTGACCTGTCTGCAATCAC
serpina3nR	ACAGTTTCGCAGACATTGGG
TIMP-1F	GGATATGCCCACAAGTCCCA
TIMP-1R	AGGACCTGATCCGTCCACAA
MMP-9F	CCAGAGGTAACCCACGTCAG
MMP-9R	TTGGAAACTCACACGCCAGA
mmu-miR-12196-5 pF	CTCCTTTCTAGCTCACCTGTGACT
mmu-miR-1946aF	AGCCGGGCAGTGGTGG
mmu-miR-1199-3 pF	TGCGGCCGGTGCTCAGT

F: forward primer; R: reversed primer.

**Table 2:** Commonly identified DEMs in the miRWalk database.

DEMs	miRNAs
Upregulated	miR-6953-5p, miR-6953-3p, miR-6956-3p, miR-201-5p, miR-7054-5p, miR-7056-3p, miR-7232-5p, miR-328-5p, miR-7053-5p, miR-6954-3p, miR-6954-5p, miR-7055-5p, miR-7048-3p, miR-6363, miR-3093-3p, miR-1199-3p, miR-6413, miR-7231-3p, miR-7055-3p, miR-6955-5p, miR-1946a, miR-6955-3p, miR-6952-3p, miR-7051-5p, miR-1194, miR-135b-5p, miR-6962-5p, miR-878-3p, miR-3060-5p, miR-206-5p, miR-873a-5p, miR-7054-3p, PUC2MM, miR-27b-5p, miR-7057-3p, miR-499-5p, and miR-208b-5p

Downregulated	miR-206-3p, miR-6353, miR-3106-3p, miR-5046, miR-7045-3p, miR-3618-5p, miR-1a-3p, miR-3475-5p, miR-12196-5p, miR-1247-3p, miR-296-5p, miR-467a-3p, miR-126b-5p, miR-133b-3p, miR-6900-3p, miR-6923-3p, miR-5131, miR-6390, miR-5620-3p, miR-7041-5p, miR-5098, let-7b-3p, miR-7016-3p, miR-6982-3p, miR-6241, miR-1969, miR-7241-5p, miR-1a-1-5p, miR-5623-3p, and miR-6387

DEMs: differentially expressed miRNAs.

**Table 3:** Kyoto Encyclopedia of Genes and Genomes (KEGG) results for the top 10 hub genes.

Term	Description	Count	*P* value
mmu04668	TNF signalling pathway	3	0.001935577
mmu05146	Amoebiasis	3	0.002226864
mmu05132	Salmonella infection	2	0.049703118

## Data Availability

The datasets used and analyzed during the current study are available from the corresponding author on reasonable request.
